# Involvement of heat shock protein 27 in the susceptibility of KT human breast cancer cells to UVC and interferon lethality

**DOI:** 10.3892/etm.2012.696

**Published:** 2012-09-04

**Authors:** XIAO-BO TONG, KAZUKO KITA, SHI-PING CHEN, XIA JIANG, SHIGERU SUGAYA, WEN-LI JING, SHU-FENG ZHANG, NOBUO SUZUKI

**Affiliations:** 1 Department of Physiology, Chengde Medical University, Chengde 067000, P.R. China;; 2 Department of Environmental Biochemistry, Graduate School of Medicine, Chiba University, Chiba 260-8670, Japan

**Keywords:** heat shock protein 27, glucose-regulated protein 78, breast cancer, human cell, ultraviolet ray C, interferon-β

## Abstract

Revealing the key molecules regulating the stress-response pathways in human cells is an intriguing problem. Chaperones, such as glucose-regulated protein 78 (GRP78) and heat shock protein 27 (HSP27), are important molecules for protecting the viability of human cells; however, it remains to be further clarified whether the molecules differentially modulate cellular responses to various types of stressors, such as DNA-damaging ultraviolet ray C (principally 254-nm wavelength, UVC) and cytocidal cytokine interferons. In the present study, the human breast cancer cell lines KT and MCF-7 were examined for GRP78 and HSP27 expression following exposure to UVC and human interferon-β (HuIFN-β). The KT cells demonstrated a higher sensitivity to both UVC and HuIFN-β lethality than MCF-7 cells. The cellular expression levels of GRP78 in KT cells, assessed by western blot analysis, were approximately 2-fold higher than that in MCF-7 cells, while the expression of HSP27 in the KT cells was 20% of the expression in the MCF-7 cells. Decreased resistance to UVC lethality was observed in *GRP78* siRNA-transfected KT cells. In addition, *HSP27* cDNA transfection of KT cells resulted in an increased resistance to UVC lethality. The cDNA-transfected KT cells showed an increased viability against HuIFN-β, compared with that of empty vector-transfected cells. By contrast, KT cells pretreated with HuIFN-β and irradiated with UVC demonstrated an increased resistance to UVC lethality, in association with increased levels of HSP27 expression. Thus, HSP27 may control the survival response pathways to both UVC and HuIFN-β in the human cells examined.

## Introduction

Whether chaperones play a role in the resistance of human cancer cells to anticancer agents such as radiation and chemical compounds is a valuable question. Previously, we found that glucose-regulated protein 78 (GRP78) and heat shock protein 27 (HSP27) have a protective role against ultraviolet ray C (UVC) lethality in RSa cells, which are an established human cell line with high UVC sensitivity (1,2). Compared with the RSa-derived UVC-resistant cell line AP^r^-1, RSa cells expressed lower amounts of GRP78 and HSP27. Using RSa and AP^r^-1 cells transfected with siRNA and/or cDNA for *GRP78* and *HSP27*, cellular amounts of these chaperones were suggested to be causally associated with cellular susceptibility to UVC lethality.

In addition to RSa cells, we previously established KT cells, which are a UVC-sensitive human cell line (3). Notably, both RSa and KT cells also show high sensitivity to cell proliferation inhibition by human interferon-β (HuIFN-β) (3). Coordination between cellular sensitivity to UVC and HuIFN was observed in various human cell lines, but the mechanisms underlying the coordination remain unclear (4–6). However, cellular chaperone metabolism may be the key for clarifying these mechanisms. Notably, UVC-sensitive cells, pretreated with HuIFN-β and then irradiated with UVC, become resistant to UVC lethality (5,6); however, the role of the chaperones in the HuIFN-β effect has not been studied.

In the present study, the cellular amounts of GRP78 and HSP27 were examined in KT cells. KT cells are derived from breast cancer tissue (3), therefore we used MCF-7 cells, another breast cancer-derived cell line, to compare the expression levels of the chaperones as well as to examine the UVC and HuIFN-β susceptibility of these cells.

## Materials and methods

### Cells and culture conditions

KT cells and their UVC and HuIFN-β susceptibility have been previously investigated (3). MCF-7 human breast cancer cells (HTB-26) were obtained from the American Type Culture Collection (Manassas, VA, USA). KT and MCF-7 cells were cultured in Eagle’s minimal essential medium (Nissui, Tokyo, Japan) supplemented with 5% (v/v) calf serum (Invitrogen, Carlsbad, CA, USA) and 10% (v/v) fetal bovine serum (Roche Diagnostics GmbH, Mannheim, Germany), respectively. All cells were grown at 37°C in a humidified atmosphere containing 5% CO_2_ and subcultured upon reaching semi-confluency.

### UVC irradiation

UVC was generated from a 6-W National germicidal lamp (Matsushita Electronic Industrial, Co., Osaka, Japan). The UVC intensity was 1.0 J/m^2^/sec, which was measured by the UV radiometer, UVR-254 (Tokyo Kogaku Kikai, Co., Ltd., Tokyo, Japan). The cells were irradiated with UVC immediately after the medium was removed and then reincubated for an appropriate time, as previously described (7). Mock-irradiated cells were treated in the same manner but without irradiation.

### HuIFN-β treatment

HuIFN-β was purchased from PeproTech, Inc. (Rocky Hill, NJ, USA). Twenty-four hours after plating the cells in dishes, HuIFN-β was added to the medium of each dish and cultured as described elsewhere (8).

### Cell survival assays

The sensitivity of cells to UVC lethality was measured using a colony survival assay, as previously reported (2). Briefly, cells were plated in 100-mm dishes (1×10^3^ cells/dish) and irradiated with UVC at the indicated doses 24 h after plating. Fourteen days after UVC irradiation, colonies were stained and their numbers were estimated. The survival fraction at each dose was expressed as a percentage of the mock-irradiated cell number. *D*_0_, which is the UVC dosage required to reduce the colony survival from any point in the exponential portion of the surviving fraction curve to 37% of that point, was calculated.

Cell sensitivity to HuIFN-β was measured by the viability assay using methylthiazol tetrazolium (MTT), as previously described (1). Briefly, cells were plated into 96-well plates (1.3×10^4^ cells/well) and incubated for 3 days. After incubation, living cells were assayed by incubation with 0.5 mg/ml MTT (Sigma, St. Louis, MO, USA) for 4 h, followed by measurement of absorbance. Cell viability was calculated by the absorbance as a percentage of the mock-treated cells (1).

### Immunoblotting analysis

Cells were washed twice with phosphate-buffered saline and whole cells were lysed with SDS sample buffer and boiled for 5 min. Whole-cell lysates were separated in a 10% SDS gel and immunoblotting analysis was performed as described elsewhere (2). GRP78 protein was detected with mouse anti-GRP78 monoclonal antibody (SPA-827; dilution, 1:1,000; StressGen, Victoria, BC, Canada), and HSP27 protein was detected with mouse anti-HSP27 monoclonal antibody (mH3; dilution, 1:1,000) (9,10). The antigen-antibody complexes were incubated with horseradish peroxidase-conjugated anti-mouse IgG antibody (Amersham Biosciences, Buckinghamshire, UK), followed by the ECL system reaction (Amersham Biosciences). Protein levels of actin were also analyzed as the loading control, using mouse anti-actin antibody (dilution, 1:20,000; ICN Biomedicals, Inc., Costa Mesa, CA, USA). The intensity of protein signals was quantified by the MultiGauge Ver2.2 image analyzing software (Fuji Photo Film Co., Ltd., Tokyo, Japan) and expressed as a value relative to actin.

### Construction of expression vectors

Full-length human *HSP27* cDNA was prepared according to a previously reported method (2). Briefly, the cDNA was ligated into pQE-30 plasmid (Qiagen, Germantown, MD, USA) using the *Sac*I and *Pst*I restriction sites. The construction was confirmed by sequencing and was then digested with *Eco*RI and *Hin*dIII. To construct a His-tagged HSP27 (His-HSP27) expression vector [His-HSP27/pcDNA3.1(-)], the digested fragment was gel-purified and then ligated into pcDNA3.1(-) (Invitrogen), using the *Eco*RI and *Hin*dIII restriction sites. The construct was confirmed by sequencing.

### Plasmid transfection

Cells grown to 60–80% confluency in 35-mm dishes were transiently transfected with the indicated expression plasmids using Lipofectamine™ LTX reagent (Invitrogen) and Plus™ reagent (Invitrogen) according to the manufacturer’s instructions.

### GRP78 knockdown

Duplex small interfering RNA (siRNA) with Stealth modification against human *GRP78* (*GRP78* siRNA) was synthesized based on the nucleotide sequence (Invitrogen), as previously described (11). Stealth RNAi negative control duplex (NC siRNA), with a GC content similar to that of the above Stealth RNAi, was used as a negative control. The siRNAs (100 nM) were transfected into cells for 6 h using Lipofectamine™ 2000 (Invitrogen) according to the manufacturer’s instructions, as described elsewhere (12). Two days after transfection, the cells were harvested and used for immunoblotting analysis and cell survival assays.

### Statistical analysis

Statistical analysis was performed using the Student’s t-test with the StatView software (version 4.5; Abacus Concepts Inc., Berkeley, CA, USA).

## Results

### Discrepancy in UVC sensitivity between MCF-7 and KT cells

**Figure 1:**
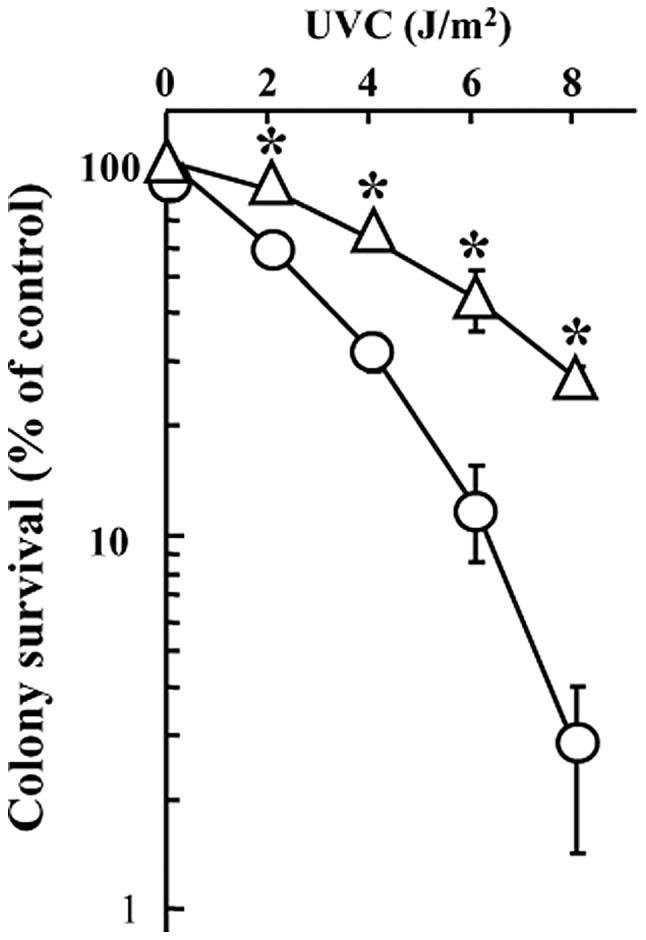
Cell survival after UVC irradiation in KT and MCF-7 cells. Cells were mock or UVC irradiated, and their survival was measured by the colony survival assay. The surviving fraction was plotted according to the UVC dosage for KT (○) and MCF-7 (Δ) cells. Data representing the average percentage of colony numbers relative to the mock-irradiated control cells are presented as mean ± SD of 3 independent experiments. ^*^P<0.05, MCF-7 vs. KT cells. UVC, ultraviolet ray C.

The sensitivity of MCF-7 and KT cells to UVC-induced cell death was examined by the colony survival assay. KT cells showed a higher UVC sensitivity than MCF-7 cells, with *D*_0_ values of 2.3 and 5.5 J/m^2^, respectively (Fig. 1).

### Discrepancy in expression levels of GRP78 and HSP27 in MCF-7 and KT cells

**Figure 2:**
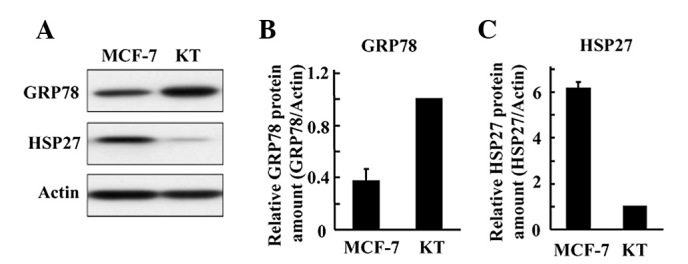
Expression levels of GRP78 and HSP27 in MCF-7 and KT cells. Protein levels were examined by (A) western blot analysis, and relative expression levels of (B) GRP78 and (C) HSP27 were analyzed. Actin was used as the loading control. Data are means ± SD of 3 independent experiments. GRP78, glucose-related protein 78; HSP27, heat shock protein 27.

To evaluate chaperones associated with the high sensitivity of KT cells to UVC lethality, the expression levels of GRP78 and HSP27 were examined in MCF-7 and KT cells by western blot analysis. The GRP78 expression level was higher in KT than in MCF-7 cells (Fig. 2A and B), while the HSP27 expression level was lower in KT than in MCF-7 cells (Fig. 2A and B).

### Relationship between GRP78 expression and UVC-sensitivity of KT cells

**Figure 3:**
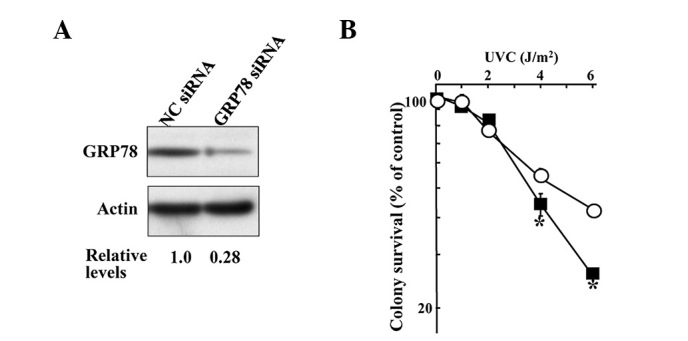
Effect of *GRP78* siRNA transfection on UVC sensitivity of KT cells. (A) Seventy-two hours after transfection with *GRP78* siRNA and NC siRNA, cells were lysed and protein levels of GRP78 and actin were analyzed by western blot analysis. Relative levels represent GRP78 amounts after normalization with actin amounts. (B) Seventy-two hours after transfection with *GRP78* siRNA (▪) and NC siRNA (○), survival of the cells after UVC irradiation was measured by the colony survival assay. Data represent the percentage of colony numbers relative to the mock-irradiated cells. The data are the mean ± SD of 3 independent experiments. ^*^P<0.05, *GRP78* siRNA-transfected vs. NC siRNA-transfected cells. NC siRNA, RNAi negative control duplex; UVC, ultraviolet ray C; GRP78, glucose-related protein 78.

To investigate the role of the high levels of GRP78 expression in KT cells compared with MCF-7 cells, we knocked down the expression of GRP78 in KT cells by transfection with siRNA for *GRP78*. The transfectants with *GRP78* siRNA demonstrated lower GRP78 protein expression than control KT cells transfected with NC siRNA (Fig. 3A). Compared with the NC siRNA-expressing cells, at UVC irradiation of up to 2 J/m^2^ the *GRP78* siRNA-expressing cells showed the same sensitivity to UVC-induced cell death, although they showed higher sensitivity when the UVC irradiation was higher than 4 J/m^2^ (Fig. 3B).

### Involvement of HSP27 expression in cellular susceptibility to UVC of KT cells

**Figure 4:**
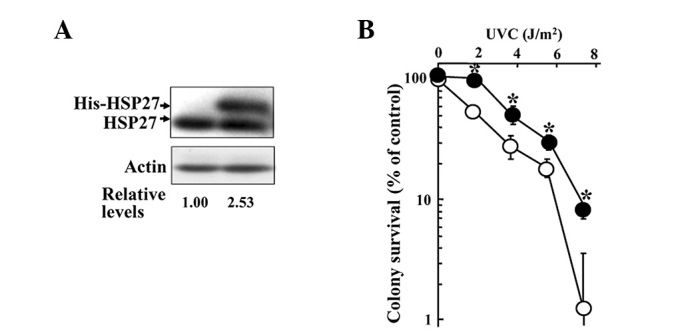
Effect of HSP27 protein overexpression on UVC sensitivity of KT cells. (A) Forty-eight hours after transfection with His-HSP27/pcDNA3.1(-) and pcDNA3.1(-), cells were lysed and protein levels of His-HSP27, HSP27 and actin were analyzed by western blot analysis. Relative levels represent the sum of exogenous His-HSP27 and endogenous HSP27 amounts after normalization with actin amounts. (B) Forty-eight hours after transfection with His-HSP27/pcDNA3.1(-) (•) and pcDNA3.1(-) (○), survival of the cells after UVC irradiation was measured by the colony survival assay. Data represent the percentage of colony numbers relative to the mock-irradiated cells. Data are the mean ± SD of 3 independent experiments. ^*^P<0.05, His-HSP27/pcDNA3.1(-)-transfected vs. pcDNA3.1(-)-transfected cells. HSP27, heat shock protein 27; UVC, ultraviolet ray C.

To investigate whether low levels of HSP27 are causally associated with the high UVC susceptibility of KT cells, we induced HSP27 overexpression in KT cells by transfection with His-HSP27/pcDNA3.1(-). The transfectants exhibited higher expression of the His-HSP27 protein than the control KT cells that were transfected with an empty vector (Fig. 4A). In addition, the colony survival assay demonstrated that the His-HSP27-expressing cells showed lower sensitivity to UVC-induced cell death than the control cells (Fig. 4B).

### Involvement of HSP27 in cellular HuIFN-β susceptibility of KT cells

**Figure 5:**
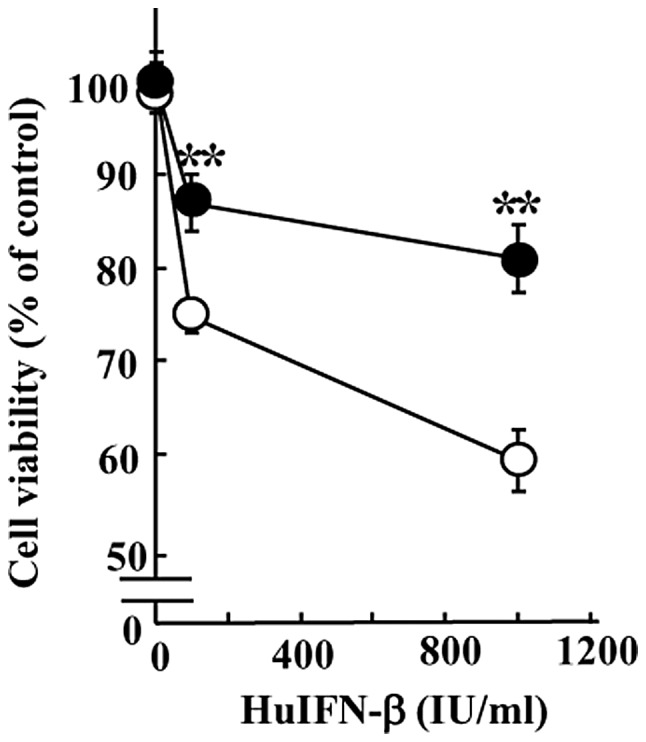
Effect of HSP27 protein overexpression on HuIFN susceptibility of KT cells. Forty-eight hours after transfection with His-HSP27/pcDNA3.1(-) (•) and pcDNA3.1(-) (○), cells were treated with HuIFN-β at the indicated dose, and 48 h later cell viability was analyzed by the MTT assay. Data represent the percentage of viability relative to the mock-treated cells. Data are the mean ± SD of 3 independent experiments. ^**^P<0.005, His-HSP27/pcDNA3.1(-)-transfected vs. pcDNA3.1(-)-transfected cells. HSP27, heat shock protein 27; HuIFN-β, human interferon-β.

To evaluate the involvement of HSP27 in HuIFN susceptibility of KT cells, we used the MTT assay to determine the viability of His-HSP27-expressing cells 48 h after HuIFN-β treatment in comparison with control cells transfected with an empty vector. The former cells showed increased viability compared with the latter cells (Fig. 5). The viability of His-HSP27-expressing cells after treatment with HuIFN-β was similar to MCF-7 cells treated with the same interferon dose (Table I).

### Involvement of HSP27 in HuIFN-β-induced resistance of KT cells to UVC lethality

**Figure 6:**
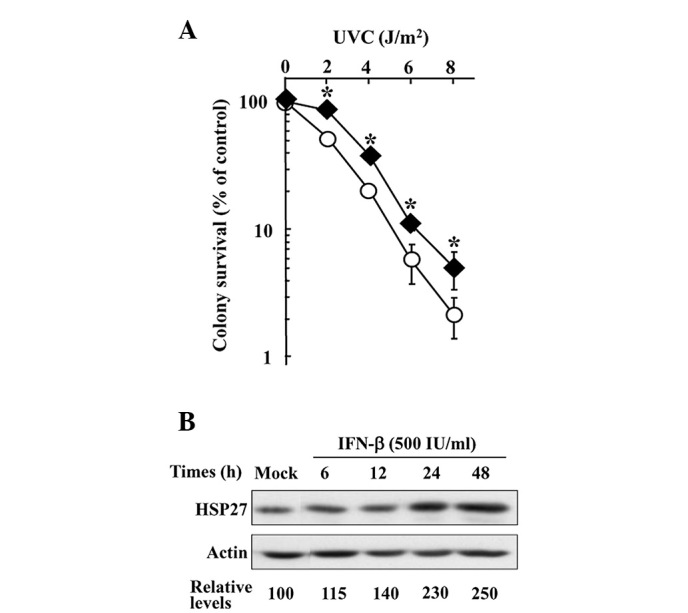
Effect of HuIFN-β pretreatment on UVC sensitivity and the expression levels of HSP27 in KT cells. (A) Cells were pretreated with HuIFN-β (50 IU/ml) for 24 h, and then cell survival after UVC irradiation was measured by the colony survival assay. (B) After a 24-h treatment with IFN-β (500 IU/ml), cells were lysed and protein levels of HSP27 and actin were analyzed by western blot analysis. Relative levels represent HSP27 amounts after normalization with actin amounts. ^*^P<0.05, HuIFN-β-treated vs. mock-treated cells. HSP27, heat shock protein 27; HuIFN-β, human interferon-β; UVC, ultraviolet ray C.

The susceptibility of HuIFN-β-pretreated KT cells to UVC lethality compared with mock cells without HuIFN-β pretreatment was tested using the colony survival assay. Following pretreatment with 50 IU/ml HuIFN-β for 24 h, the cells showed slightly higher colony survival after UVC irradiation compared with the mock cells (Fig. 6A).

To determine whether the expression levels of HSP27 increase in HuIFN-β-treated KT cells, comparative analysis of the expression between HuIFN-β and mock treatment was performed by western blot analysis (Fig. 6B). The HSP27 expression levels in cells treated with 500 IU/ml HuIFN-β for >24 h increased by >2-fold compared with the mock-treated cells (Fig. 6B).

## Discussion

In the present study, the relative roles of HSP27 and GRP78 chaperones in the cellular susceptibility to UVC and HuIFN lethality were investigated between two human breast cancer cell lines, MCF-7 and KT cells.

It appears likely that GRP78 plays a role in the susceptibility of human cells to DNA damaging agents, e.g., a protective role against UVC-induced cell death, possibly via enhancement of the DNA-nucleotide excision repair ability (1). Previously, we suggested that the protective role of GRP78 is associated with its constitutively expressed levels rather than the UVC-induced expression levels, and that GRP78 is involved in regulating the metabolism of nucleotide excision repair enzymes. KT cells, examined here, demonstrated higher levels of GRP78 expression than MCF-7 cells (Fig. 2), although the former cells were more sensitive to UVC lethality than the latter cells (Fig. 1). Thus, GRP78 expression levels do not appear to be associated with the UVC-sensitivity discrepancy between KT and MCF-7 cells, and the involvement of GRP78 expression in the UVC resistance of KT cells was unclear. However, KT cells transfected with *GRP78* siRNA showed a decreased capacity of colony survival after UVC irradiation greater than 4 J/m^2^ compared with the NC siRNA transfectants (Fig. 3B). Therefore, GRP78 expression levels may be partially associated with the UVC resistance of KT cells, although the resistance is low.

HSP27 appears to be another chaperone responsible for the UVC resistance of human cells (2). The UVC resistance may be due to increased DNA-repair capacity, including removal of UVC-induced damage, thymine dimers (6-4) and photoproducts (2). As HSP27-overexpressing KT cells showed increased levels of colony survival (Fig. 4A and B), HSP27 may play a role in the increased UVC resistance of KT cells via the modulation of DNA-repair mechanisms. However, the UVC-induced DNA-repair capacity of KT cells appears to be normal (3). Recently, we observed that Annexin II, an HSP27-binding protein, confers UVC-resistance to human cells (13,14). Therefore, it is necessary to further investigate the mechanisms underlying the involvement of HSP27 and/or its binding protein in UVC resistance.

Yonekura *et al* (15) reported that HuIFN-γ, but not HuIFN-α, downregulates HSP27 expression and suppresses the negative regulation of cell death in cells derived from oral squamous cell carcinoma. However, in our study, HuIFN-β treatment was found to upregulate HSP27 expression in KT cells (Fig. 6B), possibly leading to the increased resistance of KT cells to HuIFN-β (Fig. 5) and UVC lethality (Fig. 6A). Previously, we reported that HuIFN-α treatment results in a similar increased UVC resistance of human UVC-sensitive cells (5). Therefore, HuIFN-α and -β may act differently than HuIFN-γ at the point of cell survival modulation against UVC. In addition, HSP27 may play a role in cellular HuIFN-β susceptibility in association with increased resistance of the cells to HuIFN-β lethality (Table I).

## Figures and Tables

**Table I:** Effects of HSP27 overexpression on HuIFN-susceptibility of KT cells.

		Viability (% of control) after HuIFN-β treatment	
		HuIFN-β (IU/ml)	
Cells	Transfection	100	1000
MCF-7	Mock	91.6	78.1
KT	Mock	81.0	67.2
KT	pcDNA3.1(-)	74.9	67.3
KT	His-HSP27/pcDNA3.1(-)	90.1	84.2

HSP27, heat shock protein 27; HuIFN-β, human interferon-β.
